# Fatty Acids from Membrane Lipids Become Incorporated into Lipid Bodies during *Myxococcus xanthus* Differentiation

**DOI:** 10.1371/journal.pone.0099622

**Published:** 2014-06-06

**Authors:** Swapna Bhat, Tye O. Boynton, Dan Pham, Lawrence J. Shimkets

**Affiliations:** Department of Microbiology, University of Georgia, Athens, Georgia, United States of America; University of Groningen, Groningen Institute for Biomolecular Sciences and Biotechnology, Netherlands

## Abstract

*Myxococcus xanthus* responds to amino acid limitation by producing fruiting bodies containing dormant spores. During development, cells produce triacylglycerides in lipid bodies that become consumed during spore maturation. As the cells are starved to induce development, the production of triglycerides represents a counterintuitive metabolic switch. In this paper, lipid bodies were quantified in wild-type strain DK1622 and 33 developmental mutants at the cellular level by measuring the cross sectional area of the cell stained with the lipophilic dye Nile red. We provide five lines of evidence that triacylglycerides are derived from membrane phospholipids as cells shorten in length and then differentiate into myxospores. First, in wild type cells, lipid bodies appear early in development and their size increases concurrent with an 87% decline in membrane surface area. Second, developmental mutants blocked at different stages of shortening and differentiation accumulated lipid bodies proportionate with their cell length with a Pearson's correlation coefficient of 0.76. Third, peripheral rods, developing cells that do not produce lipid bodies, fail to shorten. Fourth, genes for fatty acid synthesis are down-regulated while genes for fatty acid degradation are up regulated. Finally, direct movement of fatty acids from membrane lipids in growing cells to lipid bodies in developing cells was observed by pulse labeling cells with palmitate. Recycling of lipids released by Programmed Cell Death appears not to be necessary for lipid body production as a *fadL* mutant was defective in fatty acid uptake but proficient in lipid body production. The lipid body regulon involves many developmental genes that are not specifically involved in fatty acid synthesis or degradation. MazF RNA interferase and its target, enhancer-binding protein Nla6, appear to negatively regulate cell shortening and TAG accumulation whereas most cell-cell signals activate these processes.

## Introduction

Lipid bodies are carbon storage organelles found in most eukaryotic organisms. They consist of triacylglycerides (TAGs) surrounded by a single phospholipid layer and associated proteins. In eukaryotes, lipid bodies are dynamic organelles that regulate lipid metabolism, membrane trafficking, signaling, and protein degradation. Alterations in TAG metabolism influence the risk of developing diabetes and other metabolic diseases in humans [1]. Therefore, biogenesis and regulation of lipid bodies has become an area of intense research. TAGs are rarely found in prokaryotes, where the most prevalent carbon storage molecules include polyhydroxyalkanoates, trehalose, and glycogen. Nevertheless, lipid bodies occur in several Actinomycetes and a few Proteobacteria species such as *Acinetobacter calcoaceticus* ADP1 [2]. In these prokaryotes, TAG synthesis generally occurs in response to high carbon to nitrogen ratio [3].

Recently, lipid bodies were discovered in *Myxococcus xanthus*, a member of the δ-Proteobacteria that forms fruiting bodies containing spores [4]. *M. xanthus* development is induced by carbon limitation [5], [6] suggesting that TAGs are produced from existing cellular material. Lipid bodies first appear 6 h after development is induced by amino acid deprivation, attain their largest size at 18 h, and disappear in the mature spore. It is not clear how *M. xanthus* lipid body synthesis is mediated or regulated. Disruption of genes encoding proteins associated with mature lipid bodies did not compromise lipid body production [4].

DK1622 (wild type) cells observed after nutrient deprivation on a solid surface contain lipid bodies of various sizes that can be visualized by Nile red staining. These lipid bodies comprise a substantial portion of the cell volume. The chemical composition of the lipid body lipids is known in detail and consists primarily of TAGs, some containing ether-linked fatty alcohols instead of ester-linked fatty acids [4]. As development is induced by carbon limitation, where does the carbon for lipid body production originate? Unlike *Bacillus* endospore formation where the spore is formed inside a mother cell, *Myxococcus* sporulation is an encystment in which the long, thin rod-shaped cells shorten then round up to become spherical myxospores [7]. Cylindrical cells about 7 µm in length and 1 µm in diameter produce spherical spores roughly 1.8 µm in diameter. Excluding the thick cortex and spore coat layers, the diameter of the membrane-bound spore interior is about 1 µm [8]. Thus, the membrane surface area declines from 23.6 µm^2^ to 3.1 µm^2^. On theoretical grounds, membrane phospholipids could serve as the principle carbon source for TAGs within lipid bodies with little biochemical complexity to the conversion. Two alternate possibilities also exist. Fatty acids could be salvaged from cells undergoing Programmed Cell Death (PCD) or they could be synthesized *de novo* using carbon reserves from elsewhere in the cell.

In this work, we show that lipid body production is closely coupled with reduction in cell length during development. When growing cells were pulse labeled with palmitate, the label appeared in the membrane during growth then shifted into lipid bodies during development. The results point to a novel method of producing TAGs from phospholipids.

## Results and Discussion

### Lipid bodies appear as cell length diminishes in WT cells

**Figure 1:**
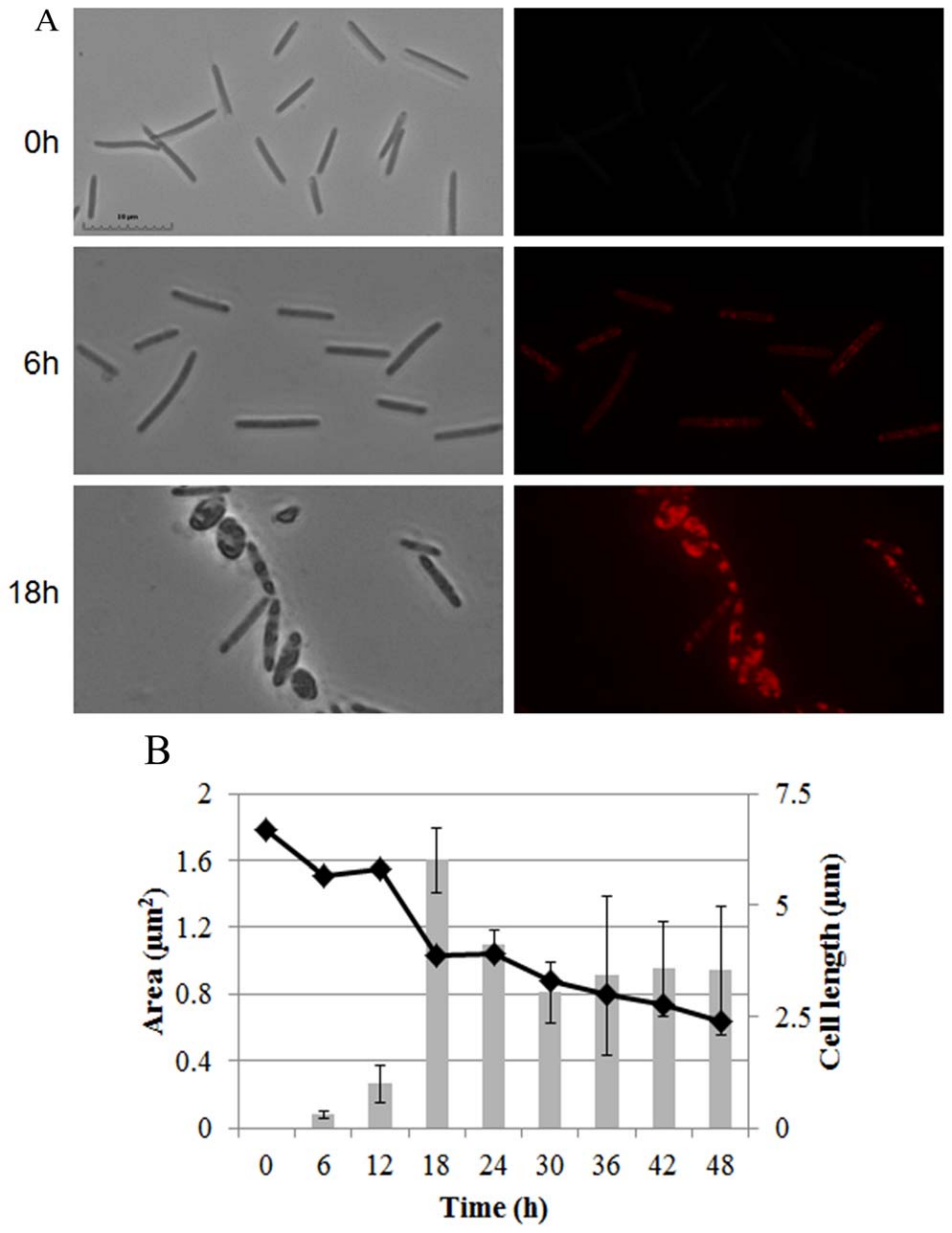
Lipid body production in WT cells during development. (A) DK1622 cells stained with the lipophilic dye Nile red at times indicated during development. Phase (Left), fluorescence (Right). Bar is 10 µm. (B) Lipid bodies were quantified by measuring the average cross sectional area stained with Nile red using at least 30 cells (grey bars). Cell length was measured using phase contrast images of 30 randomly chosen cells (filled diamonds). At 48 h, the cells are a nearly equal mixture of long, peripheral rods and spherical myxospores.

Lipid body size was quantified using Nile red stained wild type DK1622 cells [4]. Vegetative cells grown in a rich medium (0 h) have no lipid bodies (Figure 1A). 6 h after starvation initiates development, several small lipid bodies appear close to the membrane. Over the next 12 h, lipid bodies increase in size and number. By 18 h, the peak of lipid body production, the area of the cell stained with Nile red is roughly 20% the cross-sectional area of the cell. Cell shortening begins soon after initiation of development in WT cells and coincides temporally with the appearance of lipid bodies. By 18 h, a few cells have become spherical while the cylindrical cells are about 40% shorter (Figure 1B). Lipid bodies then decline in size and number after 18 h (Figure 1B) before finally disappearing completely in mature spores. The coupling of diminishing cell length with lipid body accumulation suggests the use of internal carbon sources as a reservoir for TAG building blocks. Another possibility is the recycling of lipids released by cells undergoing PCD. A small amount of lysis occurs by 18 h [9].

We attempted to distinguish between these possibilities by examining the phenotype of a *fadL* mutant. FadL, a porin that facilitates the movement of fatty acids across the outer membrane, is essential for fatty acid catabolism in bacteria [10]. In *Escherichia coli*, long chain fatty acid uptake is abolished in mutants lacking *fadL* [11]. The phenotype of *M. xanthus fadL* mutant LS3125 (MXAN7040) is strikingly similar to that of the wild type, proficient in both development and lipid body formation. Thus, it appears as if the majority of carbon used to synthesize TAGs is produced from internal reservoirs generated from the shrinking cell.

### Peripheral rods remain long and are devoid of lipid bodies

Within the fruiting body, both cell shortening and lipid body production are observed in 80% of cells destined for PCD, and 10% of cells that sporulate [9]. Peripheral rods comprise the remaining 10% of developing cells and rarely enter the fruiting body [12]. While the function of peripheral rods remains unknown, they appear to express different proteins compared with cells inside the fruiting body [12]. Peripheral rods do not make lipid bodies [4]. If lipid bodies are derived from the existing cell membrane and thus coupled to the shrinking cell, then peripheral rods might be expected to remain long. We determined the lengths of cells lacking lipid bodies. The average length of 30 cells remained the same through three different time points, 18 h (7.0 µm±1.4), 30 h (7.2 µm±1.4), and 48 h (7.3 µm±1.7). Thus, developing cells devoid of lipid bodies remain long throughout development. This observation is consistent with the idea that cell shortening and lipid body production are coupled processes.

### Genes for fatty acid synthesis are down regulated during development

**Figure 2:**
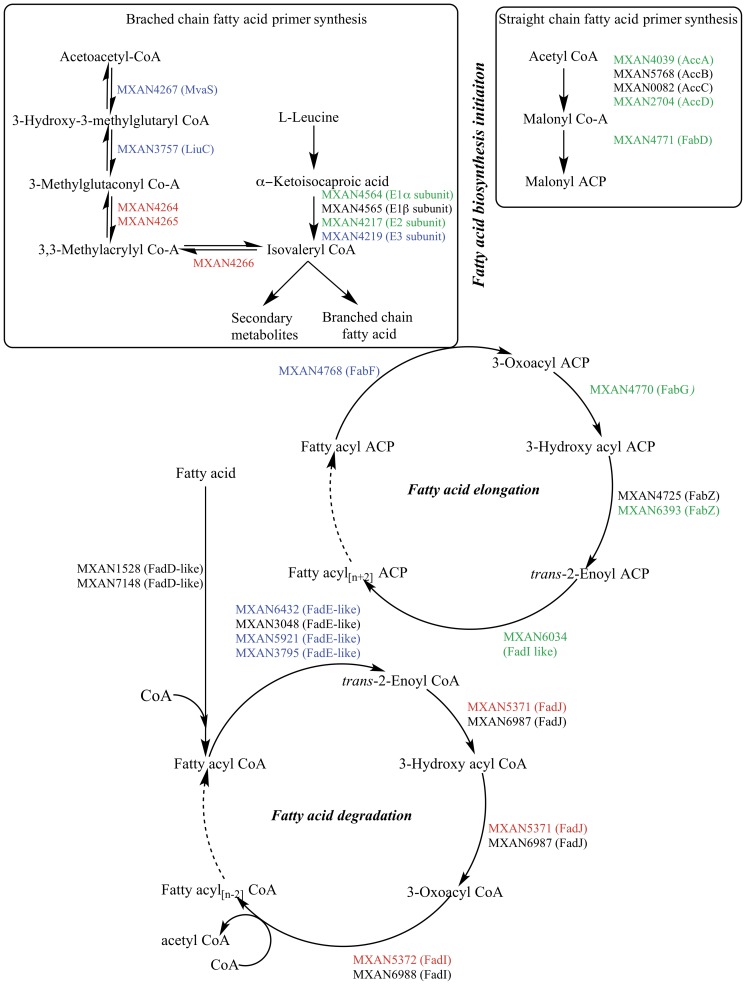
*M. xanthus* fatty acid metabolic pathways were analyzed at the transcriptional level using available microarray data from developing cells [13]. The pathway information and gene annotations were obtained from the Kyoto Encyclopedia of Genes and Genomes (KEGG) pathway database. Genes that are down-regulated (≥1.5 fold), up-regulated (≥1.5 fold) or unchanged during development are shown in green, red, and black, respectively. Blue denotes genes that were not on the microarray. A dashed arrow between two compound names implies that the two names represent the same compound in different stages of polymerization or depolymerization.

To determine whether the internal carbon reservoir for lipid body production involves existing fatty acids or instead utilizes *de novo* fatty acid synthesis, we examined the expression of fatty acid biosynthetic genes during development. If TAGs are derived from membrane phospholipids, one might expect a decline in transcription of fatty acid biosynthesis genes. Gene expression was quantified using published microarray data from developing wild type cells (Accession number GSE9477) [13]. The *M. xanthus* fatty acid pathways assigned by the Kyoto Encyclopedia of Genes and Genomes (KEGG) Pathways database are shown in figure 2 [14], [15]. Proteins proposed to mediate each reaction are shown next to the arrows as both MXAN numbers and, where known, 4-letter protein names.

*M. xanthus* straight chain fatty acid synthesis begins with an activation sequence that produces malonyl acyl carrier protein (Malonyl ACP) (Figure 2, upper right). All genes required for straight chain primer synthesis are down-regulated with respect to vegetative cells (MXAN4039, 2.1-fold; MXAN2704, 5.7-fold; MXAN5768, 1.5-fold, MXAN0082, 1.3-fold, and MXAN4771, 1.9-fold). For branch chain fatty acids, the enzyme branched chain keto acid dehydrogenase (BCKAD) makes primers that are then elongated. Here, L-leucine is converted to isovaleryl CoA, the primer for the *iso* odd family of fatty acids including *iso*15:0 the major fatty acid. MXAN4564 encodes the E1α subunit of BCKAD and is down-regulated 12.7-fold while MXAN4565 (E1β subunit) and MXAN4217 (E2 subunit) are down-regulated 1.4-fold and 1.5-fold, respectively (Figure 2, upper left). The E3 subunit of BCKAD was not present on the microarray and thus undetermined. *M. xanthus* has an additional, novel pathway for making isovaleryl CoA [16]–[18]. Three genes identified in this pathway are up-regulated during development (MXAN4264, 2.9 fold; MXAN4265, 2.0-fold; MXAN4266, 3.8-fold). While the upregulation of the latter pathway is counter to the results with BCKAD, this pathway is thought to primarily function in secondary metabolite production. For elongation, both straight and branch chain primers are extended two carbons at a time using the fatty acid synthase cycle until they reach full length (Figure 2, center circle). Genes within the cycle are down-regulated (MXAN4770; 1.5-fold, MXAN4725, 1.4-fold, MXAN6393, 1.6-fold, and MXAN6034, 1.4-fold).

The most common and energetically effective pathway for fatty acid catabolism is β-oxidation. Here, two carbon atoms are removed from the fatty acid with each turn of the cycle. In general, genes involved in β-oxidation are up-regulated during development. In *E. coli*, the first step involves esterification of a fatty acid molecule to a CoA moiety by FadD. The KEGG database predicts MXAN1528 as a possible FadD homolog. However, a BLASTP search using the *E. coli* K12 FadD sequence suggested MXAN7148 as the closest homolog. Whereas transcription of MXAN1528 does not change, transcript levels of MXAN7148 increased 1.3-fold (Figure 2, bottom circle). In the next step, acyl CoA is oxidized to enoyl CoA by FadE. The KEGG database identified 3 homologs. A fourth, MXAN3795, is the closest homolog identified by BLASTP using *E. coli* K12 FadE. Only MXAN3048 is on the array and expression increases 1.3-fold. The subsequent steps in the fatty acid degradation cycle are catalyzed by FadI and FadJ. *M. xanthus* contains two homologs of each. MXAN5371 (FadJ) and MXAN5372 (FadI) are up-regulated 3.0-fold and 2.3-fold respectively whereas the other pair is not up-regulated. Thus, it appears that genes involved in the degradation of fatty acids are expressed during development, consistent with the observation that lipid bodies disappear in mature spores.

In summary, the data call into question the hypothesis that the fatty acid content of lipid bodies is synthesized *de novo* during development. Microarray data show that fatty acid biosynthesis genes are down regulated. These data are consistent with the idea that preformed fatty acids are used to make TAGs. The largest source of fatty acids is the pool of membrane phospholipids, which are of limited utility in a shrinking cell. TAGs provide an uncharged, non-toxic intermediate that can be stored until needed for carbon and energy during spore maturation.

### Most developmental mutants have reduced lipid bodies

**Figure 3:**
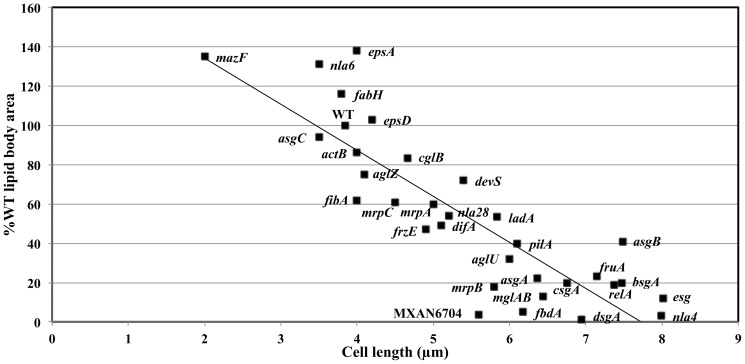
Lipid body area correlates with cell length in developmental mutants. Lipid body production at 18table 1.

If cell shortening and lipid body production are obligately coupled, then mutants defective in cell shortening should also be defective in lipid body production. We examined over 30 developmental mutants at 18 h, the peak of lipid body production in wild type cells. The mutant set includes most of the commonly studied developmental mutants known to have defects in fruiting body morphogenesis, myxospore differentiation, or both. Lipid body area per cell was averaged from 30 cells at 18 hours of development. The mutants show a wide range of variability from 0% to nearly 140% of wild type levels (Table 1). Average cell length was also calculated for these cells (Table 1). The mutant set reflects a continuum in shape change including those that fail to decrease cell length, those that initiate cell shortening, and those that shorten and ultimately sporulate despite delayed timing. A plot of lipid body area vs. cell length at 18 h shows that lipid body area increases as cell length declines over much of the mutant set (Figure 3). The line describing the best fit to the entire mutant collection passes through the standard deviations for WT cells. The Pearson's correlation coefficient between cell length and lipid body area for the entire mutant collection is 0.76. These results argue that for most mutants, cell length is proportional to lipid body content regardless of the stage at which the mutants are blocked.

**Table 1:** Lipid body production and average cell length in developmental mutants.

Strain	%WT lipid body area/cell^a^	Cell length (µm)^b^
*actB*	86.0±27.3	4.0±0.8
*aglU*	32.0±0.8	6.0±1.0
*aglZ*	76.0±33.9	4.1±2.0
*asgA*	22.0±10.1	6.4±1.4
*asgB*	41.0±10.1	7.5±1.1
*asgC*	94.0±11.1	3.5±1.5
*bsgA*	20.0±13.2	7.5±2.5
*cglB*	76.0±19.5	4.7±1.2
*csgA*	20.0±1.0	6.8±1.4
*devS*	72.0±19.3	5.4±0.8
*difA*	49.0±9.6	5.1±0.9
*dsgA*	1.0±0.8	7.0±1.2
*epsA*	138.0±37.3	4.0±0.8
*epsD*	111.0±25.3	4.2±1.2
*esg MXAN4265*	11.0±9.6	8.0±2.1
*fabH*	123.0±22.6	3.8±0.5
*fbdA*	5.0±4.7	6.2±0.9
*fibA*	61.0±10.0	4.3±0.7
*fruA*	23.0±6.0	7.2±1.7
*frzE*	47.0±10.0	5.0±1.7
*ladA*	53.3±27.0	5.8±1.2
*mazF*	135.0±11.1	2.0±0.5
*mglAB*	13.0±11.0	6.4±1.5
*mrpA*	60.0±9.0	5.0±0.9
*mrpB*	18.0±8.0	5.8±1.4
*mrpC*	61.0±23.1	4.5±0.9
*MXAN6704*	3.6±3.5	5.6±1.4
*nla28*	54.0±10.7	5.2±1.4
*nla4*	2.0±2.3	8.0±2.0
*nla6*	133.0±40.0	3.5±0.6
*pilA*	40.0±10.0	6.1±2.1
*relA*	19.0±13.0	7.4±1.7
Wild type	100.0±10.8	3.6±1.2

a The average lipid body area at 18 h, the peak of lipid body production in wild type cells, was calculated from 30 cells and compared with WT plus or minus the standard deviation.

b Average cell length at 18 h plus of minus the standard deviation.

The mutant collection defines at least two stages in the cell shortening/lipid body production process. The first stage is represented by mutants whose lipid body content is 40% or less of wild type and whose average cell length is 6 µm or longer at 18 hours. Most of the known signaling mutants cluster in this group including *asgA* (A-signal), *bsgA* (B-signal), *csgA* (C-signal), *dsgA* (D-signal), and *esg MXAN4265* (E-signal). All are blocked within the first six hours of development and these represent their terminal developmental phenotypes [19], [20]. The second mutant cluster shortens substantially (4–6 µm), though not quite as much as wild type, and produces fewer TAGs (40–80% of WT). Some, like *fibA* and *mrpA*, ultimately sporulate [21] though *fibA* produces fewer spores than wild type and has a reduced rate of germination [22]. Others in this cluster, like *difA* and *mrpC*, fail to sporulate and represent terminal phenotypes [23], [24].

In conclusion, most developmental mutants are defective in producing lipid bodies even though the products of the mutant genes have no direct role in lipid metabolism. Furthermore, lipid body content is proportional with cell length as if lipid bodies are derived from some portion of the shrinking cell. As the correlation coefficient is <1, there may be several different carbon sources for TAG production that vary among mutant strains. Alternately, fatty acid oxidation could also reduce the correlation coefficient in some strains because TAGs are just a temporary residence for fatty acids. The microarray results indicate that fatty acids are consumed during development, consistent with the observation that mature spores lack lipid bodies.

### Lipid body production is regulated by branched chain fatty acids and an RNA interferase

Several mutants have an altered relationship between lipid body content and cell length that differs substantially from the correlation coefficient. The genes represented by these mutants may play direct roles in regulating cell shortening and/or lipid body production. A mutant that under produces lipid bodies relative to cell length is MXAN6704. Mutants that over produce lipid bodies relative to cell length include *asgB*, *epsA*, *nla6*, and *fabH*. *mazF* is unique in that while lipid body content is proportional to cell length, the cells are unusually short at this stage of development.

*mazF* encodes an RNA interferase recognizing an 8-nucleotide sequence [25]. MazF appears to be a negative regulator of shortening since *mazF* cells are about half as long as WT cells and have a corresponding increase in lipid content. Among the MazF targets is enhancer-binding protein *nla6* mRNA [26]. The *nla6* mutant has similar though not quite as dramatic properties (Figure 3). The *nla6* mutant overproduces lipid bodies and is slightly shorter than wild type. No other genes in this mutant collection are known to be MazF targets [26]. *nla28* and *actB* transcription is activated by Nla6, but inactivation of these genes diminishes lipid accumulation rather than enhancing it so they appear not to be negative regulators (Table 1 and Figure 3) [27].

*MXAN6704* under produces lipid bodies relative to cell length and seems to encode a Gcn5-related N-acetyltransferase (GNAT). GNATs use acetyl coenzyme A to transfer an acetyl group to the primary amine of an acceptor molecule such as lysine [28]. *MXAN6704* mutants make neither fruiting bodies nor spores. While the *MXAN6704* mutant produces only 5% of the wild type level of lipid bodies, the cells shorten more than most mutants that fail to make lipid bodies (Figure 3) indicating a partial uncoupling of cell shortening from lipid body production. Protein acetylation is thought to regulate the activity of enzymes controlling carbon flow across metabolic pathways, especially the flow of acetate as it relates to fatty acid synthesis and degradation [29], [30]. One might predict that this mutant has enhanced fatty acid degradation relative to wild type.

The *fabH* mutant is one of several mutants that over produce TAGs. *fabH* has high levels of the principle fatty acid in TAGs, branched chain fatty acid *iso*15:0, due to a defect in producing straight chain fatty acids [31]. The opposite extreme is seen for *esg MXAN4265*, which is unable to produce *iso*15:0 and fails to shorten or make lipid bodies. When *iso*15:0 or a TAG called TG1 that contains an ether-linked *iso*15:0 fatty alcohol are added to *esg MXAN4265* cells, either molecule restores lipid body production at physiological concentrations concomitantly with cell shortening and sporulation [9]. TG1 and *iso*15:0 are the twin components of the E-signal whose sensory mechanisms remain unknown [9]. These results suggest an autocatalytic cycle in which cell shortening stimulates the production of E-signal to further stimulate cell shortening.

### Fatty acids in membrane lipids of growing cells become incorporated into lipid bodies during development

Since fatty acid biosynthetic genes are down regulated during development, we directly investigated the possibility that membrane phospholipids serve as the source of preformed fatty acids for lipid body lipids. Growing *Myxococcus* cells were pulse labeled with the fatty acid palmitic acid alkyne (PA^alk^). Using click chemistry [32], Alexa Fluor 488 azide was attached to the PA^alk^ in permeabilized cells prior to visualization with fluorescence microscopy. Incorporation of the fatty acid was examined during growth in CYE complex media [33], M1 defined media [34], and A1 minimal media [35]. Fatty acid incorporation as measured by fluorescence was only detected in A1. To determine the positions of membranes and lipid bodies in the micrographs, cells were also stained with Nile red.

**Figure 4:**
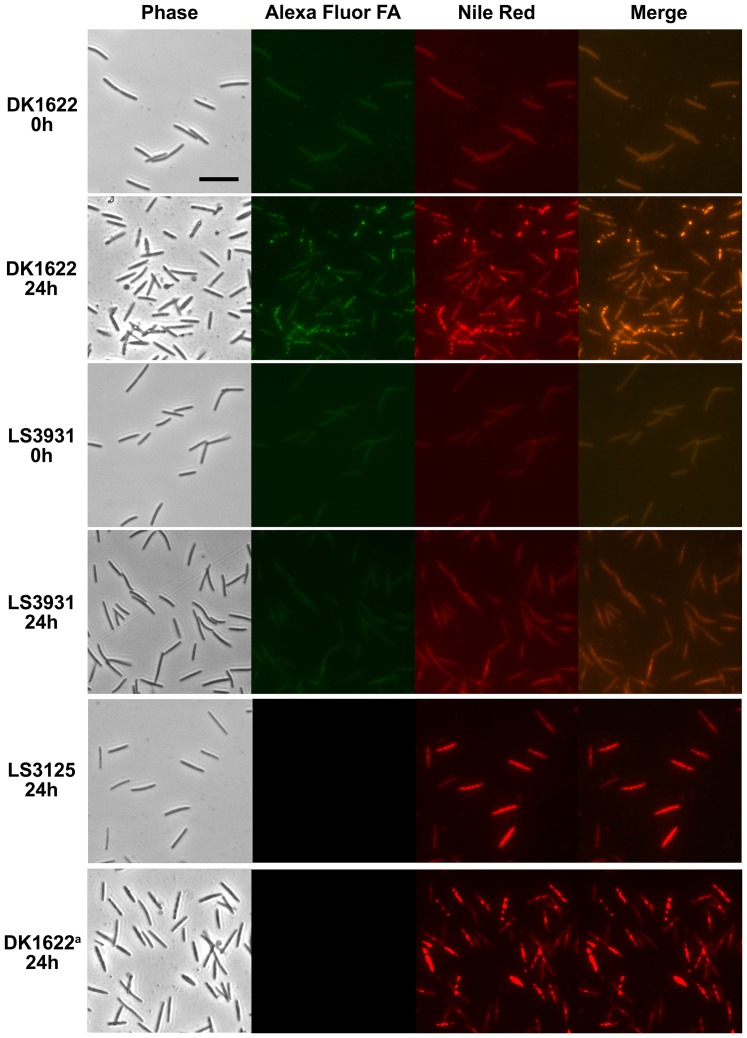
Lipid bodies are derived from membrane phospholipids. *Myxococcus* cells were grown in the presence of palmitic acid alkyne (PA^alk^) in A1 minimal medium and stained with Nile red. Click chemistry was used to attach Alexa Fluor 488 azide to PA^alk^ prior to visualization. Both Alex Fluor fatty acid and the Nile red stain are visible in membranes during vegetative growth (DK1622 0 h). The PA^alk^ was removed and cells were allowed to develop on TPM agar (DK1622 24 h). Labeled membrane lipids were incorporated into Nile red stained lipid bodies, seen in both channels and the merged images. In a strain deficient in lipid body production (LS3931), incorporation occurs into membrane lipids (LS3931 0 h) but not lipid bodies (LS3931 24 h). A *fadL* mutant (LS3125), defective in fatty acid uptake, is unable to incorporate PA^alk^ altogether. Wild-type cells grown in the absence of PA^alk^ then allowed to develop (DK1622^a^ 24 h) exhibit no fluorescence (bottom row, second panel) indicating little bleed through between channels. Scale bar is 10 µm.

In wild-type cells (Figure 4, DK1622), PA^alk^ is detected in the membranes of vegetative cells grown in A1 (0 h) as a thin layer of fluorescence that outlines each cell. The chemical form(s) was not identified, but a likely possibility is phosphatidylethanolamine, which comprises 70% of the phospholipid in *Myxococcus* [36]. By 24 h of development, PA^alk^ becomes prominent within DK1622 lipid bodies demonstrating that the TAGs are at least partially derived from the fatty acids in membrane lipids. PA^alk^ is also incorporated into membranes of a strain deficient in making lipid bodies (Figure 4, LS3931), but fully formed lipid bodies are rarely observed. No detectable signal is seen in lipid bodies from wild-type cells grown without PA^alk^ fatty acid indicating that there is no bleed through between fluorescence channels (Figure 4, last line).

Detection of exogenous PA^alk^ incorporation when cells are grown in minimal media demonstrates fatty acid uptake. As such, the possibility exists that lipid bodies are formed from exogenous material during development. Lipid bodies first arise during the early stages of PCD that ultimately claims 80% of the initial cell population. Lipids scavenged from dead cells would provide an excellent source of preformed fatty acids. To examine whether fatty acids are recycled from dead cells, incorporation of PA^alk^ was examined in LS3125, a mutant strain lacking *fadL*, an outer membrane protein necessary for fatty acid uptake. No detectable signal was seen in this mutant, though lipid bodies are clearly visible (Figure 4, LS3125). These results indicate that incorporation of exogenous cellular material from dead cells is not required for mature lipid body formation.

In summary, PA^alk^ fluorescence is always found concurrent with lipids stained with Nile red, as shown in each of the merged images. In growing cells, these are membrane lipids that surround the cell. In developing wild type cells, lipid bodies are strongly labeled from preformed fatty acids consistent with the idea that membrane phospholipids are mobilized to make TAGs as the membrane surface area declines. As the results are not quantitative, we cannot rule out the possibility that the lipid body fatty acids supplied by membrane phospholipids are augmented by some *de novo* fatty acid synthesis.

### The developmental regulon regulates the lipid body regulon

**Figure 5:**
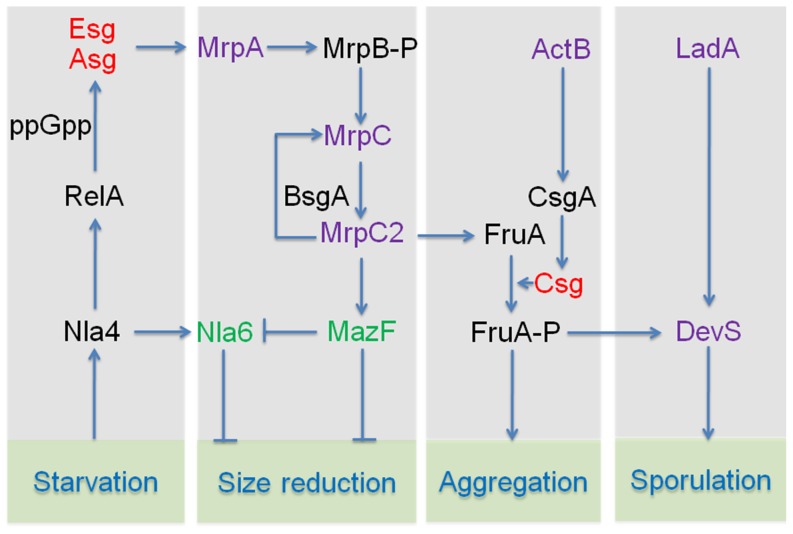
Regulation of lipid body production during *M. xanthus* fruiting body development. Mutants blocked in synthesis of proteins labeled in in black are deficient in lipid body synthesis (>40%). Those labeled in purple produce intermediate levels of lipid bodies (40–80%). Those in green produce near WT levels or higher (>80%). Red letters indicate developmental signals. Asg, A-signal; Csg, C-signal; Esg, E-signal.

Figure 5 provides a simplistic model of the lipid body/developmental regulon in *M. xanthus* illustrating the regulatory points for lipid body production. Development begins with amino acid deprivation. Nla4, an enhancer binding protein, activates expression of *relA* (synthesis of (p)ppGpp) and initiates the stringent response [37]–[39]. Both mutants exhibit little shortening and little TAG accumulation (Figure 3). Sensing starvation initiates production of the A-signal, a quorum signal composed of specific amino acids designed to determine whether the cell density is sufficient for development [40]. E-signaling also begins about this time [23], [41].

Five genes are required for A-signal production. While *asgA* (22%) and *asgB* (41%) deficient strains produce few lipid bodies relative to wild type levels, the *asgC* (94%) mutant produces nearly normal levels. These results argue that lipid body production is not dependent on the A-signal, but requires another function provided by *asgA* and *asgB*. AsgA is a hybrid histidine kinase/response regulator [42]. AsgB is a putative DNA binding protein [43]. Perhaps AsgA and AsgB directly or indirectly activate genes required for lipid body production irrespective of their role in A-signal production.

A-signaling, or one of the Asg proteins [23], and the E-signal, a mixture of fatty acid *iso*15:0 and ether triacylglyceride TG1 [9], initiates expression of the *mrpAB* operon encoding a two-component system [44]. MrpA encodes a histidine protein kinase that presumably phosphorylates response regulator MrpB (Figure 5). The situation may be more complex since inactivation of each gene yields different phenotypes. The *mrpB* mutation is more severe, blocking aggregation, sporulation, and lipid body production (Figure 3). The *mrpA* mutation blocks sporulation but has a more modest impact on cell shortening and lipid body production.

MrpB-P goes on to activate expression of the transcription factor *mrpC*, essential for development [23]. MrpC autoactivates its own expression and induces expression of *mazF* and *fruA.* MrpC binding sites are observed in the promoter regions of all three genes [25], [45], [46]. MrpC and FruA regulate transcription of many genes involved in sporulation [23], [46], [47]. Loss of Lon protease, referred to as BsgA for its role in B-signal production [48], [49], leads to dramatic reduction in lipid bodies. BsgA is thought to activate MrpC by removing 33 amino acids from the N-terminus, though the reaction has never been demonstrated *in vitro* [45]. *mrpC*, *bsgA*, and *fruA* mutants under produce lipid bodies, though *mrpC* produces more TAGs than the other two (Figures 3 and 5). MazF is an RNA interferase that degrades enhancer binding protein *nla6* mRNA [26]. Nla4 activates transcription of *nla6* and can be considered a positive regulator of shortening and TAG production since *nla4* mutants fail to do either (Figure 3) [27].

*actB* encodes a transcriptional activator of the *act* operon leading to increased expression of *csgA* [50]. *csgA* produces the C-signal, which is essential for both aggregation and sporulation [51]. C-signaling activates response regulator FruA, a response regulator and transcriptional activator of many morphogenesis genes [52]. The method of FruA activation by C-signaling is presumably phosphorylation, though the cognate histidine kinase has not been identified. Curiously, *csgA* mutants have a more severe phenotype than *actB* mutants, even though *csgA* appears to lie downstream of *actB* (Figures 3 and 5). A *csgA* mutant fails to aggregate, sporulate, or produce lipid bodies whereas the *actB* mutant aggregates and produces nearly normal levels of lipid bodies (84%; Figure 3 and Table 1), but doesn't sporulate well [53]. Aggregates are formed as the C-signal levels rise, but even higher C-signal levels are required to induce sporulation [54], [55]. Although ActB increases C-signaling, *actB* mutants already produce one-fourth of the WT level of CsgA, which is sufficient to induce aggregation [56]. This level of C-signaling in *actB* cells also seems sufficient for the initial phase of cell shortening and lipid body production. The *mglAB* mutant is nonmotile and consequently defective in C-signaling, which requires cell alignment for efficient transmission of C-signal [57]. *mglAB* is also severely defective in lipid body production, comparable to *csgA* (Table 1).

Sporulation within fruiting bodies is triggered by DevTRS, proteins of unknown function whose production is activated in part by FruA and in part by a second LysR-type activator, LadA, which also binds to the *dev* promoter region [58]–[60]. *ladA* and *devS* mutants produce comparable and relatively high levels of lipid bodies suggesting that mutations in late developmental genes have only limited reduction of lipid bodies.

In summary, there is no single point in the developmental program where lipid body production becomes activated to the full extent. TAG synthesis is inhibited by mutations in most developmental genes even though these genes are not directly related to lipid metabolism. These include most of the early cell signal-producing genes, which abolish lipid body production. Taken together, these results suggest that development consists of a series of checkpoints designed to precisely couple shortening with TAG production.

## Conclusions

*M. xanthus* development is initiated by carbon limitation. Unexpectedly, lipid bodies containing TAGs become a major, though temporary, development-specific product that is consumed during spore maturation. Where does the carbon for TAG synthesis originate? Two major reservoirs of carbon may be available to sporulating cells, an extracellular one generated by PCD and an internal one generated by cell shrinkage. PCD eliminates 80% of the cells, and fatty acids could be recycled by extracellular phospholipases and incorporated into prespores. *M. xanthus* produces many lipolytic enzymes that could facilitate recycling, but deletion of their genes had little effect on spore yield [61]. Furthermore, mutation of *fadL*, which encodes a porin required for fatty acid assimilation, did not disrupt development or diminish lipid body production. Clearly, lipid bodies can be produced without recycling extracellular lipids.

As there is a >80% decrease in cell volume and membrane surface area during differentiation, carbon reserves from the shrinking cell could easily be mobilized into TAGs. This possibility is supported by a temporal relationship between diminishing cell length and lipid body production in wild type cells. Furthermore, there is a strong correlation between cell length and lipid body content among mutants blocked during various stages of development. The shrinking cell could produce TAGs by either *de novo* fatty acid synthesis or by recycling of fatty acids in membrane phospholipids. Genes involved in fatty acid biosynthesis are down regulated, so we instead focused our attention on lipid conversion. Membrane lipids that were pulse labeled with palmitate during growth served as a source of fatty acids for lipid bodies. While these results illustrate the movement of fatty acids from membrane lipids to TAGs, the experiments were not quantitative and we cannot rule out the possibility that mobilization is supplemented with some fatty acid synthesis and/or some recycling of extracellular lipids. We predict the existence of complex machinery designed to shorten the cell and mobilize membrane phospholipids to TAGs as part of a pathway in which fatty acids are eventually degraded to produce mature spores. The enzymes involved in synthesizing TAGs from phospholipids remain unknown. *M. xanthus* contains a single diacylglycerol acyltransferase gene, but deletion of this gene did not eliminate TAG production ([4] and unpublished).

## Materials and Methods

### Bacterial strains and growth condition

Table 2 lists the bacterial strains used in this study and their sources. *M. xanthus* strains were grown in CYE broth [1% Bacto casitone (Difco), 0.5% yeast extract (Difco), 10 mM 4-morpholinepropanesulfonic acid (MOPS) (pH 7.6), and 0.1% MgSO_4_)] at 32°C with vigorous shaking. Kanamycin and Bacto agar (Difco) were added to CYE at final concentrations of 50 µg/ml and 1.5% respectively. Development was induced on TPM agar plates [10 mM Tris HCl, pH 7.6, 1 mM KH(H_2_)PO_4_, pH 7.6, 10 mM MgSO_4_, 1.5% agar (Difco)].

**Table 2:** *M. xanthus* strains used in this study.

Bacterial strain	Genotype	Reference or source
DK1622	WT	[65]
DK3260	*dsgA429*	[66]
DK4398	*asgB480*	[67]
DK5057	*asgA476*	[68]
DK5061	*asgC767*	[69]
DK5614	*fabH*	[31]
DK6204	Δ*mglAB*	[70]
DK10410	Δ*pilA*	[71]
DK10603	Δ*actB*	[50]
DK11063	*fruA*	[52]
DK11209	Δ*devS*	[72]
LS313	*cglB*	Lawrence Shimkets
LS1191	*esg MXAN4265*	[9]
LS1193	*mazF*	Lawrence Shimkets
LS2208	Δ*fibA*	Lawrence Shimkets
LS2225	*fbdA*	[73]
LS2442	Δ*csgA*	Lawrence Shimkets
LS2702	*MXAN6704*	This study
LS3931	Δ*csgA*, *socA*	Lawrence Shimkets
MS10	*relA*	[74]
AG328	*nla28*	[37]
AG304	*nla4*	[37]
AG306	*nla6*	[37]
MPVlysR	*ladA*	[60]
MXH1777	Δ*aglU*	[75]
MXH2265	Δ*aglZ*	[76]
RGM252	*bsgA302*	[48]
SW810	Δ*epsA*	[77]
SW813	Δ*epsD*	[77]
SW2802	Δ*mrpB*	[78]
SW2807	Δ*mrpA*	[78]
SW2808	Δ*mrpC*	[78]
SW600	Δ*frzE*	[79]
YZ601	Δ*difA*	[24]

### Strain construction

*magellan*-4 transposon mutagenesis was used for isolating new fruiting body deficient mutants [62]. Fruiting body deficient strains containing a *magellan*-4 insertion were then backcrossed to *M. xanthus* DK1622 by electroporation of 1 µg genomic DNA or by generalized transduction with phage Mx4 [63]. Insertion regions were identified by cloning and sequencing [64]. LS2702 contains a *magellan*-4 insertion within *MXAN6704.*

### Nile red staining

*M. xanthus* strains were grown to a density of 5×10^8^ cells/ml then resuspended in 100 µl dH_2_O to a final density of 5×10^9^ cells/ml. Aliquots of 10 µl were spotted on TPM agar and incubated for various times. Lipid body staining was carried out as described by Hoiczyk et al with modifications [4]. A 0.5 mg/ml stock solution of Nile red (Sigma Aldrich) prepared in 100% ethanol was diluted in dH_2_O to a final concentration of 1.25 µg/ml and added directly on top of cells developing on TPM agar. The plates were incubated for 2 h at 32°C. Cells were resuspended in a drop of TPMF buffer [TPM buffer containing 10% ficoll], and examined with a fluorescence microscope (Leica Microsystems, DM5500B). Digital images were obtained using a QICAM FAST 1394 camera (Q Imaging systems, Compix Inc.).

The fluorescence images were digitally altered using Simple PCI (Hamamatsu Corporation) to remove background noise, improve contrast, and measure cell length and fluorescence intensity. Average lipid body area and cell length were calculated from 30 randomly chosen cells.

### Fatty acid incorporation

For incorporation of labeled fatty acids, *Myxococcus xanthus* cells were grown to a density of 1×10^8^ cells/ml in A1 minimal media [0.5% potassium aspartate, 0.5% sodium pyruvate, 0.05% (NH_4_)_2_SO_4_, 0.2% MgSO_4_, 0.125 mg/ml spermidine, 0.1 mg/ml each asparagine, isoleucine, phenylalanine and valine, 0.05 mg/ml leucine, 0.01 mg/ml methionine, 1 µg/ml cobalamin, 10 µM FeCl_3_, 10 µM CaCl_2_, 1 mM KH(H_2_)PO_4_ pH 7.6, and 10 mM Tris HCl pH 7.6] [35] containing 100 µM palmitic acid alkyne (Cayman Chemical).

For visualization, click chemistry was used to attach Alexa Fluor 488 azide to the alkyne moiety of the fatty acid after growth or development. 1×10^9^ cells were permeablized by resuspension in 1 ml TPM containing 2% paraformaldehyde and incubated with gentle shaking for 1 h at room temperature. Cells were then harvested at 13,000 rpm for 1 min and resuspended in 1 ml phosphate buffered saline (PBS), pH 7.4, with 0.02% Tween 20. Cells were next harvested and washed in 1 ml TGE buffer (50 mM glucose, 20 mM Tris pH 7.6, 10 mM EDTA), resuspended in 1 ml TGE containing 10 µg/ml lysozyme, and incubated for 10 min at room temperature. Cells were washed in 1 ml PBS containing 2% BSA, then resuspended in 500 µl Click-iT reaction (Life Technologies) containing 440 µl Click-iT buffer, 10 µl 100 mM CuSO_4_, and 50 µl 1 mg/ml Alexa Fluor 488 azide. Cells were incubated for 30 min at room temperature in the absence of light. After the reaction was complete, cells were harvested and resuspended in 1 ml TPM containing 2% BSA, stained with Nile red as previously described in this paper, and visualized using a fluorescence microscope (Leica Microsystems, DM5500B).
